# *Mycobacterium mageritense* Pulmonary Disease in Patient with Compromised Immune System

**DOI:** 10.3201/eid1703.101279

**Published:** 2011-03

**Authors:** R. Gordon Huth, Barbara A. Brown-Elliott, Richard J. Wallace

**Affiliations:** Author affiliations: University of Texas Southwestern Residency Programs, Austin, Texas, USA (R.G. Huth);; University of Texas Health Science Center, Tyler, Texas, USA (B.A. Brown-Elliott, R.J. Wallace, Jr.)

**Keywords:** Bacteria, tuberculosis and other mycobacteria, Mycobacterium mageritense, atypical mycobacteria, pulmonary disease, compromised immune system, letter

**To the Editor:** *Mycobacterium mageritense* is one of the rapidly growing mycobacteria (RGM). It was first isolated in Spain in 1987, described as a new species in 1997 by Domenech et al. (*1*), and first described and associated with disease in the United States in 2002 (*2*). In the 2002 report, 6 isolates were recovered from sputum, a bronchoscopy sample, a wound infection after liposuction, the blood of an immunosuppressed patient with a central catheter and sepsis, a patient with severe sinusitis, and from a wound infection in a patient who had probable osteomyelitis after fixation of an open fracture. It has since been reported as a cause of water-related skin and soft tissue infections (*3**,**4*). A study from Japan in 2007 reported recovery of *M. mageritense* from the sputum of a woman with noncaseating granulomas by transbronchial biopsy who improved without therapy (*5*). We describe a case of *M. mageritense* pneumonia in an immunocompromised patient.

In 2009, a 54-year-old woman was admitted to the hospital in Austin, Texas, with a 5-day history of upper back pain and occasional hemoptysis and yellow sputum production. She had a long history of systemic lupus erythematosus and associated nephritis and vasculitis, rheumatoid arthritis, hypothyroidism, sleep apnea, and hepatitis C infection. She was taking prednisone 15 mg/day at the time of admission.

Five months earlier, organizing pneumonia was diagnosed in the patient by computed tomography–guided lung biopsy of a pleura-based mass; special stains and cultures on tissue for acid-fast bacilli (AFB), other bacteria, and fungi were negative. She was readmitted several times over subsequent months and treated with various antimicrobial agents and corticosteroids but did not show clinical or radiographic improvement. Chest computed tomographic scan performed at admission again demonstrated bilateral lung masses and infiltrates, with new areas of necrosis. A second needle biopsy sample showed chronic inflammation with a histiocytic reaction and negative stains for AFB and fungi, but it was deemed nondiagnostic. Subsequent open lung biopsy sample showed necrotizing granulomatous inflammation with possible vascular involvement suggestive of Wegener granulomatosis.

Fite staining showed rare clusters of AFB within the granulomas. The postoperative course was complicated by a multiloculated left pleural effusion. AFB smear of pleural fluid obtained from video-assisted thoracoscopy showed 1–5 bacilli per high power field. Cultures of lung tissue and pleural fluid grew mycobacteria initially identified as *M. fortuitum* group but subsequently identified as *M. mageritense* by PCR followed by restriction enzyme analysis of the 65-kDa heat-shock protein (*hsp65*) (*6*). Results of susceptibility testing by broth microdilution are shown in the Table.

**Table:** In vitro activity of 23 isolates of *Mycobacterium mageritense*, United States, 2009*

Antimicrobial agent	No. isolates tested	MICs of current isolate, μg/mL	Intermediate breakpoint, μg/mL	MIC range, μg/mL	MIC_50_, μg/mL	MIC_90_, μg/mL	% S/I
Amikacin	23	8	32	<1–32	16	32	100
Cefoxitin	23	16	32–64	<8–256	32	64	91
Ciprofloxacin	23	0.25	2	<0.25–0.5	0.25	0.5	100
Clarithromycin†	23	8	4	1–>64	>32	>64	4
Doxycycline	22	1	2–8	0.25–>64	8	>32	50
Imipenem	22	4	8	<0.5–8	2	4	100
Linezolid	22	4	16	<2–16	4	8	100
Sulfamethoxazole	21	4	32	<2–32	8	32	100
Trimethoprim/sulfamethoxazole	6	1/19	2/38‡	<0.25/4.8– 2/38	0.5/9.5	2/38	100
Tobramycin	23	<2	8	2–64	>16	>32	30
Tigecycline	5	0.12	–§	<0.03–0.12	0.06	0.12	NA

*Includes 6 isolates previously reported (*2*). S, susceptible; I, intermediate; NA, not available. †Three days’ incubation. ‡Proposed breakpoint (*7*). §No Clinical and Laboratory Standards Institute breakpoints established for tigecycline.

Testing for Wegener granulomatosis by antineutrophilic cytoplasmic and myeloperoxidase antibody testing yielded negative results. Imipenem and amikacin were prescribed, and gradual resolution of clinical signs and symptoms was observed. Oral linezolid and trimethoprim/sulfamethoxazole were prescribed at discharge. Chest radiographs taken 4 months after the open lung biopsy showed resolution of the masses.

The isolate was a nonpigmented RGM that matched the American Type Culture Collection (Manassas, VA, USA) type strain and 10 published clinical isolates of *M. mageritense* by PCR restriction enzyme analysis of the 65-kDa *hsp* gene (*6*). By gene sequencing of region V of the RNA polymerase (*rpoB*) gene, it exhibited 99.7% identity to the GenBank type strain sequence of *M. mageritense* (acceptable interspecies relatedness for this sequence is >98.5% identity) (*8*). The most closely related species determined by using this sequence and previously submitted sequences were other *M. fortuitum* species: *M. porcinum* (94% sequence identity), *M. wolinskyi* (94%), and *M. peregrinum* (93%).

Susceptibility testing of 23 clinical isolates of *M. mageritense* from the United States previously submitted to the Mycobacteria/Nocardia Research Laboratory (University of Texas Health Science Center, Tyler, TX, USA) and identified by *hsp65* PCR restriction analysis (*6**,**7*) was performed (Table). These results confirmed the potential utility of the drugs used in this case for future cases.

*M. mageritense* has not been reported as a cause of pulmonary disease in an immunocompromised patient. However, most cases of *M. fortuitum* pneumonia were reported before the use of molecular technology for species identification. Newer species such as *M. mageritense* resemble *M. fortuitum* and would not have been differentiated without this method.

Our patient met the criteria for diagnosing nontuberculous mycobacterial lung disease as established by the American Thoracic Society and the Infectious Diseases Society of America (*9*). Her therapeutic response also supports a cause-and-effect relationship.

The identity of an RGM isolate as *M. mageritense* may be suspected by its unusual antimicrobial drug susceptibility pattern, which showed an intermediate MIC to amikacin and resistance to clarithromycin at 3 days (Table). However, definitive identification requires molecular methods. Previous studies have shown that *M. mageritense* contains an inducible erythromycin methylase gene (*erm* 40) that confers macrolide resistance (*10*). The use of molecular studies and greater attention to susceptibility patterns should enable increased recognition of *M. mageritense* as a human pathogen.
